# Association of detected depression and undetected depressive symptoms with long-term mortality in a cohort of institutionalised older people

**DOI:** 10.1017/S2045796015001171

**Published:** 2016-01-12

**Authors:** J. Damián, R. Pastor-Barriuso, E. Valderrama-Gama, J. de Pedro-Cuesta

**Affiliations:** 1 National Center for Epidemiology, Carlos III Institute of Health, Madrid, Spain; 2 Consortium for Biomedical Research in Neurodegenerative Diseases (CIBERNED), Madrid, Spain; 3 Consortium for Biomedical Research in Epidemiology and Public Health (CIBERESP), Madrid, Spain; 4 Arroyo de la Media Legua Primary Care Center, Madrid Health Service, Madrid, Spain

**Keywords:** Depression, epidemiology, mortality, nursing homes

## Abstract

**Background.:**

Studies on depression and mortality in nursing homes have shown inconclusive findings, and none has studied the role of detection. We sought to measure the association of depression with long-term all-cause mortality in institutionalised older people and evaluate a potential modification in the association by its detection status.

**Methods.:**

We selected a stratified cluster sample of 591 residents aged 75 years or older (mean age 84.5 years) living in residential and nursing homes of Madrid, Spain, who were free of severe cognitive impairment at the 1998–1999 baseline interview. Mortality was ascertained until age 105 years or September 2013 (median/maximum follow-up 4.8/15.2 years) through linkage to the Spanish National Death Index. Detected depression was defined at baseline as a physician's diagnosis or antidepressant use, undetected depression as significant depressive symptoms (score of 4 or higher on the ten-item version of the Geriatric Depression Scale) without documented diagnosis or treatment, and no depression as the absence of diagnosis, treatment, and symptoms. Constant and age-dependent hazard ratios for mortality comparing detected and undetected depression with no depression were estimated using Cox models, and absolute years of life gained and lost using Weibull models.

**Results.:**

The baseline prevalences of detected and undetected depression were 25.9 and 18.8%, respectively. A total of 499 participants died during 3575 person-years of follow-up. In models adjusted for age, sex, type of facility, number of chronic conditions, and functional dependency, overall depression was not associated with long-term all-cause mortality (hazard ratio 0.87, 95% confidence interval (CI): 0.70–1.08). However, compared with no depression, detected depression showed lower mortality (hazard ratio 0.63, 95% CI: 0.46–0.86), while undetected depression registered higher, not statistically significant, mortality (hazard ratio 1.35, 95% CI: 0.98–1.86). The median life expectancy increased by 1.8 years (95% CI: −3.1 to 6.7 years) in residents with detected depression and decreased by 6.3 years (95% CI: 2.6–10.1 years) in those undetected. Results were more marked in women than men and they were robust to the exclusion of antidepressants from the definition of depression and also to the use of a stricter cut-off for the presence of depressive symptoms.

**Conclusions.:**

The long-term mortality risk associated with depression in nursing homes depends on its detection status, with better prognosis in residents with detected depression and worse in those undetected. The absolute impact of undetected depressive symptoms in terms of life expectancy can be prominent.

## Introduction

The relationship between depression and mortality has been frequently studied. Indeed, three systematic reviews in the general population (Wulsin *et al*. 1999; Cuijpers *et al*. 2014) and in older people (Schulz *et al*. 2002) cover almost all the available literature on the issue, with compelling evidence of an increased risk of dying linked to depression. In addition, depressive symptoms have also been associated with mortality in community-dwelling elderly (Sun *et al*. 2011; White *et al*. 2015). In residential and nursing homes, some (Rovner *et al*. 1991; O'Connor & Vallerand, 1998; Barca *et al*. 2010; Kane *et al*. 2010; Drageset *et al*. 2013) but not all cohort studies (Cohen-Mansfield *et al*. 1999; Parmelee *et al*. 1992; Cuijpers, 2001; Sutcliffe *et al*. 2007) have found a positive association of depression and depressive symptoms with mortality. Most of these studies were conducted over short follow-up periods and none has properly examined the potential role of detection in the association between depression and mortality, though one study did provide some related data (Rovner *et al*. 1991). In a previous report, we found that 42% of depressed residents were undetected, corresponding to 18% of the whole institutionalised population (Damián *et al*. 2010). Accordingly, the aims of this study were to measure the association of depression with long-term all-cause mortality and evaluate a potential effect modification according to its detection status in a representative population sample residing in facilities for older people.

## Method

### Study population

This cohort study used mortality follow-up data from a baseline survey conducted from June 1998 through June 1999 in a stratified cluster sample of residents aged 65 years or older in residential and nursing homes of Madrid, Spain. We initially selected 25 public/subsidised and 30 private institutions with probability proportional to their size, and then sampled ten men and ten women from each public/subsidised facility, and 5 men and 5 women from each private facility. Due to refusal or prolonged absence, 85 residents could not be included, leading to an overall response rate of 89% (715 out of 800 sample residents). Thirty nine subjects were randomly substituted with residents of the same facility and sex, yielding a total of 754 baseline interviews. In the present study we excluded subjects with severe cognitive impairment since they were likely unable to remember or report effective depressive symptoms.

The Carlos III Institutional Review Board approved the study. Informed consent was obtained verbally and documented from all study participants or their next of kin.

### Baseline data collection

Structured questionnaires were administered by trained geriatricians to all residents, their main caregivers, and the facility physicians to collect baseline data on sociodemographic characteristics, medical history, depressive symptoms, functional dependency, and cognitive status.

Physician's diagnosis of depression was ascertained by interviewing facility physicians (or nurses in 8% of residents) with access to medical history. Antidepressants used for the preceding 7 days (code N06A of the World Health Organization Anatomical Therapeutic Chemical Classification) were noted by reviewing medical records. A ten-item version of the Geriatric Depression Scale (GDS) was administered verbally to each resident to assess depressive symptoms over the previous week without focusing on physical complaints (D'Ath *et al*. 1994). This self-report measure has shown adequate diagnostic accuracy in the institutional setting, including residents with mild to moderate cognitive impairment. The overall discrimination of the ten-item GDS was 0.86 for identifying clinical major or minor depression among nursing home patients, with sensitivity rates of 75–86% and specificity rates of 70–77% for the optimal cut-off score of 4 or higher (Shah *et al*. 1996; Jongenelis *et al*. 2005). Although the ten-item GDS has not been validated for Spanish population, translation and adaption of longer 15- and 30-item versions to the Spanish language were straightforward and showed similar psychometric properties to those of the original questionnaires (Izal & Montorio, 1993; Martínez de la Iglesia *et al*. 2002). (See supplementary material.)

Depression was defined as a physician's diagnosis of the condition, use of antidepressants, or a score of 4 or higher on the ten-item GDS. Residents with depression were considered to be detected if they had a physician's diagnosis or had been prescribed antidepressants, irrespective of depressive symptoms; whereas depressed residents were deemed to be undetected if they scored 4 or higher on the ten-item GDS, without documented diagnosis or treatment.

Chronic conditions, including cancer, obstructive pulmonary disease, arrhythmias, hypertension, ischaemic heart disease, congestive heart failure, peripheral arterial disease, stroke, diabetes, anemia, Alzheimer's disease, other dementias, Parkinson's disease, epilepsy, anxiety disorders and arthritis, were gathered from the physician's interview. Functional dependency in performing basic activities of daily living was assessed by residents or their main caregivers (if assigned, 45%) using the modified Barthel index (Shah *et al*. 1989). Residents were classified as functionally independent (100 points), mild to moderate dependency (61–99 points), and severe to total dependency (0–60 points) (Shah *et al*. 1989).

Cognitive status was evaluated using both the Short Portable Mental Status Questionnaire (SPMSQ, 0–10 errors) (Pfeiffer, 1975), which was adapted to the institutional setting, and the Minimum Data Set Cognition Scale (MDS-COGS, 0–10 points) (Hartmaier *et al*. 1994; Gruber-Baldini *et al*. 2000), which obtained an assessment from the main caregivers based on selected Minimum Data Set questions. This latter scale was used because the SPMSQ could only be administered to 61% of residents for logistic reasons. Residents were classified as normal cognition (≤2 education-adjusted SPMSQ errors and ≤1 MDS-COGS points), mild to moderate cognitive impairment (3–7 SPMSQ errors and ≤8 MDS-COGS points, or ≤7 SPMSQ errors and 2–8 MDS-COGS points), and severe cognitive impairment (≥8 SPMSQ errors or ≥9 MDS-COGS points).

### Mortality ascertainment

Mortality was ascertained by requesting data on residents’ vital status to the participating facilities and through linkage to the Spanish National Death Index, which includes all deaths registered in Spain since 1987 (Ministerio de Sanidad Servicios Sociales e Igualdad, 2014). Residents contributed follow-up time from their 1998–1999 baseline interview or age 75 years, whichever occurred later, until death, age 105 years, or 15 September 2013, whichever occurred first. We restricted follow-up to the age interval 75–104 years because there were only 33 and 2 deaths in residents aged 65–74 and ≥105 years, respectively.

### Statistical analysis

Since depressive symptoms may reflect frailty conditions near death (reverse causation bias), early deaths within the first year from the baseline interview were analyzed separately from the remaining follow-up. Hazard ratios for mortality and 95% confidence interval (CI) for residents with detected and undetected depression compared with non-depressed residents were estimated using Cox proportional hazards models with age as the time scale (Thiebaut & Benichou, 2004) and adjusted for sex, type of facility (public, subsidised or private), number of chronic conditions other than depression (0–1, 2–3 or ≥4), and functional dependency (independent, mild/moderate or severe/total). We tested for effect modifications by including interactions of detected and undetected depression with each of the above baseline covariates. Non-parametric survival curves for residents with no, detected, and undetected depression were estimated as the baseline survival functions from a depression-stratified Cox model adjusted to the overall weighted percentages of baseline confounders. To allow for non-proportional hazards over age, we fitted age-dependent Cox models with interactions of detected and undetected depression with a restricted quadratic spline function of age with knots at 80, 90 and 100 years (Hess, 1994).

We also fitted a Weibull model with different location and scale parameters for each depression group and adjusted to the overall weighted percentages of baseline confounders. This model provided similar survival curves to those obtained by non-parametric methods, but allowed estimating the absolute years of life gained and lost for residents with detected and undetected depression compared with non-depressed residents as the difference in percentiles from their estimated Weibull distributions, with 95% CI derived from delta methods (Cox *et al*. 2007).

Sensitivity analyses were performed to evaluate the robustness of the results to alternative definitions of depression and further adjustments for potential confounders. Specifically, we replicated the analyses excluding antidepressants from the definition of depression, as well as using a stricter cut-off score of 5 or higher on the ten-item GDS for the presence of depressive symptoms. In addition, although residents with severe cognitive impairment were excluded from the analyses, the association between depression and mortality was further adjusted for residual differences in cognitive functioning (unimpaired or mild/moderate impairment) in the subset of residents with available data.

Due to the complex design and the different selection probabilities (residents in public/subsidised facilities and men were oversampled) all analyses were weighted to the underlying population distribution and accounted for the effect of stratification and clustering on point and interval estimates (using Taylor-linearised variance estimation). Analyses were performed using Stata, version 13.1 (Stata Corp., College Station, Texas) and R, version 2.15 (R Foundation for Statistical Computing, Vienna, Austria).

## Results

From the 754 participants in the baseline survey, we excluded 54 residents due to insufficient information on their mortality status at the end of follow-up, 33 residents who died before 75 years of age and 76 residents with severe cognitive impairment. Thus, the final sample included 591 residents who were followed up for mortality from 75 to 104 years of age. During 3575 person-years of follow-up (median/maximum of 4.8/15.2 years), 499 participants died, including 70 deaths within the first year of follow-up. Mortality rates were higher in men, residents in public/subsidised facilities, and subjects with mild to moderate cognitive impairment, and increased steadily with age and degree of functional dependency (Table 1).

**Table 1.** Population distribution and mortality rates by age interval and baseline characteristics of residents in nursing homes of Madrid, Spain, 1998–1999 to 2013

	No. of subjects(%)*	No. of person-years	No. of deaths	Mortality rate (95% CI)†
Overall	591 (100)	3574.6	499	133.7 (121.1, 147.5)
Age interval (years)				
75–84	315 (50.9)	1272.1	120	85.3 (70.4, 104.0)
85–94	436 (76.3)	1802.5	278	148.3 (130.4, 168.6)
95–104	145 (26.3)	500.0	101	190.4 (148.4, 243.5)
Sex				
Women	320 (74.9)	2006.5	271	130.7 (115.6, 147.4)
Men	271 (25.1)	1568.1	228	143.8 (124.0, 166.4)
Type of facility				
Public	369 (52.6)	2158.8	326	150.0 (133.9, 167.7)
Subsidised	58 (7.8)	320.3	46	154.9 (110.5, 215.6)
Private	164 (39.6)	1095.5	127	112.0 (92.9, 134.8)
No. of chronic conditions				
0–1	136 (23.0)	900.7	113	132.4 (109.4, 159.8)
2–3	245 (41.8)	1601.1	202	117.4 (100.8, 136.5)
≥4	210 (35.2)	1072.8	184	159.3 (132.8, 190.2)
Functional dependency				
Independent	178 (26.0)	1336.2	145	102.1 (87.9, 118.5)
Mild/moderate	295 (54.4)	1781.7	250	133.8 (116.3, 153.6)
Severe/total	106 (19.6)	402.1	92	205.0 (149.5, 277.2)
Unknown	12			
Cognitive impairment				
Unimpaired	274 (59.4)	1870.9	223	116.4 (101.4, 133.6)
Mild/moderate	165 (40.6)	777.2	141	162.1 (129.9, 201.2)
Unknown	152			

* Unweighted counts and weighted percentages. Figures across age intervals add up to more than the overall sample size because subjects may contribute to different age intervals during follow-up.

† Weighted mortality rates per 1000 person-years and 95% CI.

**Fig. 1.**
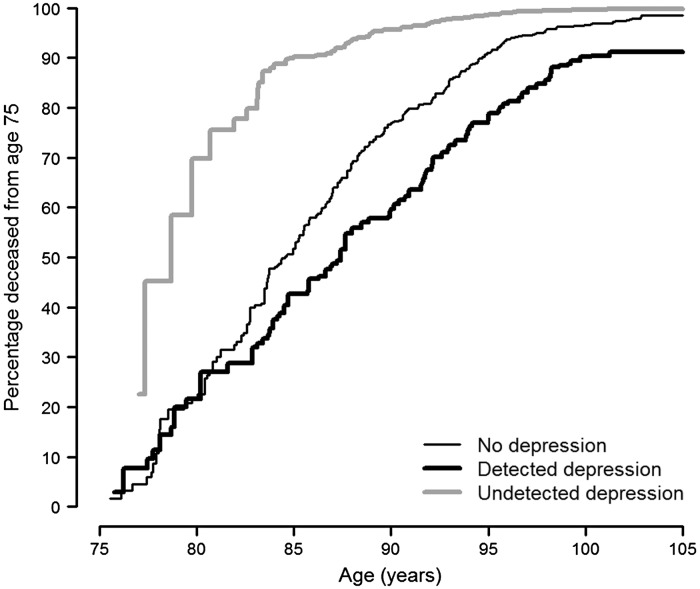
Adjusted non-parametric survival curves from age 75 for residents with no depression, detected depression, and undetected depression in nursing homes of Madrid, Spain, 1998–1999 to 2013.

**Fig. 2.**
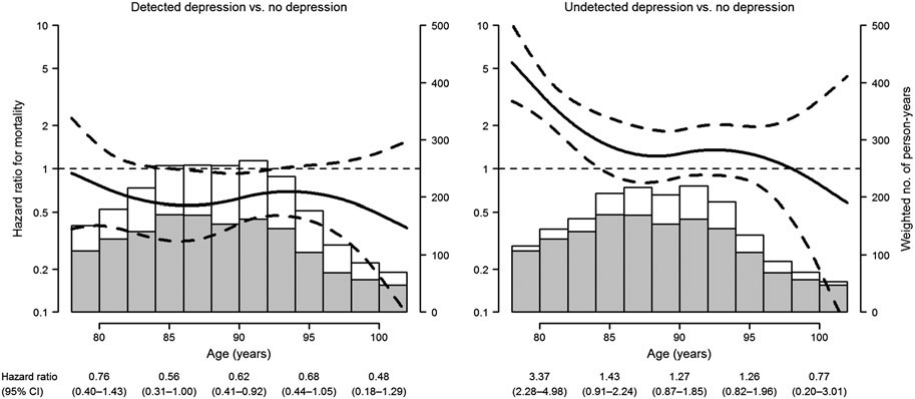
Age-dependent hazard ratios for mortality (solid curves) and 95% CI (dashed curves) in residents with detected and undetected depression compared with residents without depression in nursing homes of Madrid, Spain, 1998–1999 to 2013. The histograms represent the weighted numbers of person-years by age interval among residents with no depression (shaded bars), detected depression (left white bars) and undetected depression (right white bars).

The baseline prevalences of detected and undetected depression were 25.9 and 18.8%, respectively, adding up to an overall prevalence of depression of 44.7%. The estimates of the association of depression-related variables with mortality during the first year of follow-up were imprecise and mostly compatible with a null effect (Table 2). For the remaining long-term follow-up period, baseline physician-reported depression and antidepressant use were associated with 39 and 32% reduced risks of death in multivariate-adjusted models, respectively. In contrast, mortality risk increased by 24% among residents with baseline depressive symptoms (GDS score ≥4) compared with those without symptoms (Table 2). As a result of these opposite associations, the composite depression variable showed virtually no effect on mortality (hazard ratio 0.87, 95% CI: 0.70–1.08). However, when depression was disaggregated by its detection status, residents with detected depression registered a 37% lower mortality risk than those without depression, whereas residents with undetected depression displayed a 35% higher, not statistically significant, mortality risk (Table 2). The hazard ratio for mortality comparing undetected with detected depression was 2.14 (95% CI: 1.34–3.44). Accordingly, the adjusted survival curves display consistently lower mortality over time in residents with detected depression compared with undepressed residents (*p* for homogeneity of survival curves = 0.005) and substantially higher mortality in residents with undetected depression compared with those with no depression (*p* = 0.06) and detected depression (*p* = 0.002) (Fig. 1). The hazard ratio for detected depression *v.* no depression remained fairly stable below 1 over the entire age range (*p* for proportional hazards = 0.57), unlike that for undetected depression (*p* = 0.004), which displayed a markedly increased risk of death at ages below 85 years and a dilution in the strength of the association at older ages, mainly due to the differential depletion of residents with undetected depression (Fig. 2).

**Table 2.** Association of baseline depression-related variables with mortality risk by follow-up period among residents in nursing homes of Madrid, Spain, 1998–1999 to 2013

		First-year follow-up			2- to 15-year follow-up		
	No. of subjects (%)*	No. of person-years	No. of deaths	Hazard ratio (95% CI)†	No. of person-years	No. of deaths	Hazard ratio (95% CI)†
Physician-reported depression							
No	460 (80.0)	393.1	54	1.00 (reference)	2333.1	342	1.00 (reference)
Yes	112 (20.0)	92.7	13	0.78 (0.41, 1.46)	673.8	72	0.61 (0.44, 0.84)
Unknown	19						
Use of antidepressants							
No	520 (89.6)	439.5	63	1.00 (reference)	2680.3	381	1.00 (reference)
Yes	55 (10.4)	44.8	6	0.52 (0.18, 1.53)	325.8	35	0.68 (0.40, 1.13)
Unknown	16						
Depressive symptoms‡							
No	387 (70.2)	317.3	40	1.00 (reference)	2205.3	279	1.00 (reference)
Yes	159 (29.8)	144.2	19	1.02 (0.52, 1.98)	718.8	120	1.24 (0.97, 1.57)
Unknown	45						
Depression detection status§							
No depression	306 (55.3)	254.7	32	1.00 (reference)	1714.5	224	1.00 (reference)
Depression	235 (44.7)	203.3	27	0.73 (0.37, 1.44)	1183.6	170	0.87 (0.70, 1.08)
Detected	134 (25.9)	109.8	16	0.73 (0.33, 1.65)	792.4	87	0.63 (0.46, 0.86)
Undetected	101 (18.8)	93.5	11	0.73 (0.36, 1.49)	391.2	83	1.35 (0.98, 1.86)
Unknown	50						

* Unweighted counts and weighted percentages.

† Hazard ratios and 95% CI were obtained from Cox models splitting each subject's exposure time into the first year and the remaining follow-up and adjusted for age, sex, type of facility, number of chronic conditions other than depression and functional dependency.

‡ Score of 4 or higher on the ten-item GDS.

§ Detected depression was defined as a physician's diagnosis of depression or antidepressant use, and undetected depression as a score of 4 or higher on the ten-item GDS without documented diagnosis or treatment.

**Fig. 3.**
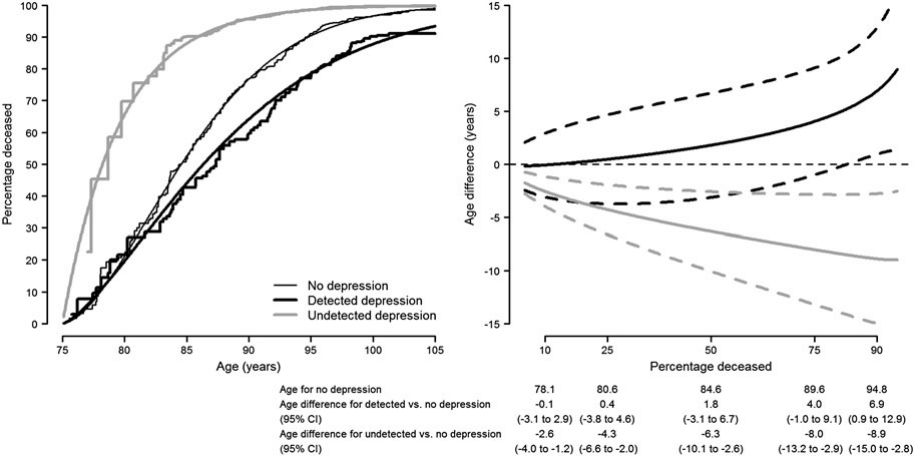
Years of life gained and lost due to detected and undetected depression among residents in nursing homes of Madrid, Spain, 1998–1999 to 2013. The left panel shows the parametric survival curves (smooth lines) obtained from a Weibull model with different location and scale parameters for each depression group, together with the corresponding non-parametric survival curves (step functions) for comparison. The right panel shows the absolute years of life gained and lost (solid curves) and their 95% CI (dashed curves) for residents with detected and undetected depression compared with non-depressed residents, as estimated from the Weibull model.

Using a Weibull model, which showed a very good fit to the non-parametric survival curves (appreciable in the left panel of Fig. 3), it was estimated that the lower, median and upper quartiles of life expectancy at age 75 for non-depressed residents were 80.6, 84.6 and 89.6 years, respectively. By the same quartiles, residents with detected depression gained 0.4, 1.8 and 4.0 years of life, whereas those with undetected depression lost 4.3, 6.3 and 8.0 years, respectively (right panel of Fig. 3). The median life expectancy decreased by 8.1 years (95% CI: 2.3–13.9 years) in residents with undetected depression compared with those detected.

**Fig. 4.**
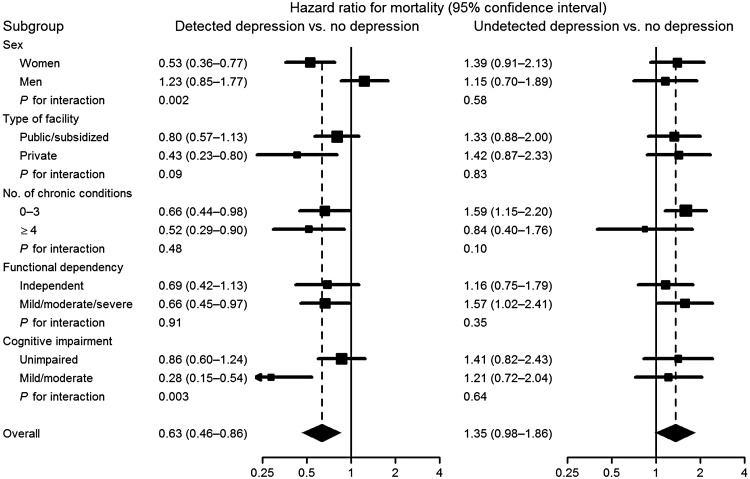
Hazard ratios for mortality (squares with area inversely proportional to the variance) and 95% CI (horizontal lines) comparing detected and undetected depression with no depression in pre-specified subgroups of residents in nursing homes of Madrid, Spain, 1998–1999 to 2013.

In subgroup analyses, the long-term beneficial effect of detected depression on mortality was higher in women (*p* for interaction = 0.002), private facilities (*p* = 0.09) and residents with mild-to-moderate cognitive impairment (*p* = 0.003). The harmful effect of undetected depression was quite homogeneous across subgroups defined by baseline covariates (Fig. 4).

In sensitivity analyses, we excluded antidepressants from the definition of depression and results were strengthened, with hazard ratios for long-term mortality comparing detected and undetected depression with no depression of 0.69 (95% CI: 0.51–0.94) and 1.50 (95% CI: 1.11–2.03), respectively. These associations did not change appreciably after adjustment for antidepressant use (hazard ratios 0.72 and 1.47). Also, we used a more stringent cut-off score of 5 or higher on the ten-item GDS, which resulted in a slight attenuation of the association between undetected depression and mortality (hazard ratio 1.24, 95% CI: 0.78–1.98). Finally, further adjustment for cognitive impairment (unimpaired or mild/moderate) in 78.9% of residents with available data yielded similar but less precise results (hazard ratios 0.59, 95% CI: 0.41–0.83, for detected depression and 1.43, 95% CI: 0.93–2.20, for undetected depression).

## Discussion

The association between depression and mortality has been consistent in most studies of community-dwelling older people but less clear in the case of the institutionalised population. However, this study found somewhat conflicting results, with a null overall association. In addition, physician-reported depression was associated with lower mortality. In-depth examination of the potential confounding effect of additional variables, such as number of medicines and several diseases, led to no appreciable change in the findings. Nonetheless when our analysis focused on depressive symptoms, we found an increase in risk in residents with symptoms. The analyses were repeated using a stricter GDS cut point of ≥5 (over 10), thus increasing specificity and decreasing sensitivity. This led some individuals to change from undetected to non-depressed with an expected dilution of the association – which in no way suffices to change our initial conclusion. A higher specificity can achieve less false positives but at the expense of increasing the probability of false negatives. In line with our results false negatives are a worse consequence because subjects can remain unattended and we show this can increase their mortality risk.

When the depressed group was broken down into detected and undetected, undetected individuals displayed a higher mortality than did those with no depression. In addition, it is nevertheless striking that the risk would be lower among those with detected depression than it was in the non-depressed group. Although we are unable to furnish an adequate explanation for this, we believe that depression detection might be part of a more general mechanism which links closer care and supervision, greater attention, responsiveness, etc. to lower mortality. In view of the fact that residents for whom detection was easier would share a better prognosis, adjustment was made for the main prognostic variables but the results remained unchanged. Detection may prove difficult in older persons, particularly in nursing homes where the quiet resident can be overlooked (Fenton *et al*. 2004). One might also speculate that, once residents have been assigned a depression diagnosis (and a treatment) and have assimilated it, this could trigger motivational changes in their attitude towards better self-care, compliance with treatments (depression and other conditions), and increased participation in activities (preventive, rehabilitative and social). In brief, detection could help break the vicious cycle of depression leading to negative conduct in self-care/activities/behaviours, which in turn leads to illness and ultimately to a return to a depressed mood.

We wish to highlight the importance of including the non-depressed group in the analytical framework not only because its absence yields a notable loss of information, but also because the non-depressed state is a part of the natural history involving detection: non-depressed, depressed but undetected and depressed but detected. In addition, the finding of non-depressed group showing a somewhat higher risk compared with detected, though admittedly incidental, is worthy of further investigation because it might be closely related to relevant aspects of care in these facilities.

For the purposes of this study, subjects using antidepressants were deemed to be depressed and detected. Admittedly, this choice is open to discussion because antidepressant use may be indicated for conditions other than depression; albeit this condition was the predominant reason for prescription in our study (63.1% of residents with prescribed antidepressants had physician's diagnosis of depression). Thus, we performed sensitivity analyses excluding antidepressants from depression definition and findings were even more marked.

According to our results it is possible that detection of depression be preventive only in women. This is in agreement with a finding on subthreshold depression in older community residents (Hybels *et al*. 2002). Men are usually more reluctant to admit or assimilate the diagnosis, leading to worse resources for resilience or coping (Hinton *et al*. 2006; Murray *et al*. 2006). In fact, the general pattern (of lower mortality in individuals with detected depression) was not confirmed in men, among whom depression detection did not seem to make a notable difference (Fig. 4).

Among the most comparable studies, Drageset *et al*. (2013) recently reported depression scores associated with 5-year mortality in a sample of cognitively intact residents from 30 nursing homes in Norway. Another of the few nursing-home-based studies to measure mortality with a long follow-up (even if undertaken in a single facility) failed to find an association between depressed affect, as evaluated by nurses, and mortality, though some association was observed in the short term (1 year) (Cohen-Mansfield *et al*. 1999). It should be noted that we found no association in the 1-year follow-up in the present study. The effects of detection/non-detection are discernible with the passage of time, and indeed ours is the only study to give an adequate description of how the hazard ratio changes with time. In primary care, Bogner *et al*. (2012) found that persistent depressive symptoms were related to mortality and demonstrate that repeated assessment of depression may be particularly important in preventing excess mortality associated to depression in older adults. We speculate that results even more favourable would be expected in nursing home population.

Potential pathways in the association of depression and mortality are multiple and complex. Some of them are discussed below.

Rovner *et al*. (1991) suggested some mechanisms, including insomnia, poor nutrition due to anorexia, immobility from psychomotor retardation and altered immune function. Cuijpers (2001) also mentioned immune function and added interference with patients’ motivation towards recovery. Somewhat similar factors are mentioned in Kane *et al*. (2010) (poor self-care, less physical activity and immune system functioning). Kiecolt-Glaser & Glaser (2002) review the evidence on the role of immune function, adding a simple but important message, the interaction of depression with ageing on enhancing risks for morbidity and mortality. This would be particularly relevant in frail elders.

### Strengths and limitations

Our study was conducted on a representative sample of individuals living in institutions (enhancing external validity), was based on a substantial sample size (limiting random error), and collected information on most of the relevant variables (limiting confounding and allowing for assessment of effect modification). Even so, some degree of depression misclassification is possible, in that we did not follow a structured assessment based on DSM-IV or ICD-10 criteria. Nonetheless we think that the group scoring 4 or more in the GDS-10 is likely to include not only a majority of depression cases as determined by that clinical criteria, but also a number of subclinical or subthreshold cases (i.e., a condition in which a person has depressive symptoms, but does not meet the criteria for a depressive disorder), likewise found to be serious and chronic (Beekman *et al*. 2002) and associated with mortality as well (Cuijpers *et al*. 2013).

Thus, taking into account our results, perhaps patients scoring 4 or more GDS-10 symptoms should merit the label of depression. At any rate, one of the main messages derived from our findings is that depressive symptoms, not declared as depression by physicians, may lead to higher mortality. As for the outcome, we believe that some deaths might not have been identified, something that would eventually generate non-differential misclassification and, in general, lead to dilution of measured associations. Finally, we recommend that these results be generalised only to subjects without severe cognitive impairment.

### Conclusions

The long-term all-cause mortality risk associated with depression depends on the condition's detection, with a better prognosis in those cases where it is detected. Had detection played a causal role, the absolute numbers of years of life lost due to undetected depression that our study estimates may be remarkable. Regarding implications on the care of nursing homes, it would seem imperative to adopt a proactive approach to the detection and treatment of depression because, whatever its causal role vis-à-vis mortality, what is undeniable is that depression leads to avoidable suffering. We endorse the recommendation that GDS be used for subjects who are not cognitively impaired, followed by initiation of treatment and monitoring of the outcome (Boyle *et al*. 2004), and the Cornell Scale for Depression in Dementia for subjects with cognitive impairment (Alexopoulos *et al*. 1988).

## Supplementary material

For supplementary material accompanying this paper visit http://dx.doi.org/10.1017/S2045796015001171. click here to view supplementary material

## References

[ref1] Alexopoulos GS, Abrams RC, Young RC, Shamoian CA (1988). Cornell scale for depression in Dementia. Biological Psychiatry 23, 271–284. 3337862 10.1016/0006-3223(88)90038-8

[ref2] Barca ML, Engedal K, Laks J, Selbaek G (2010). A 12 months follow-up study of depression among nursing-home patients in Norway. Journal of Affective Disorders 120, 141–148. 19467560 10.1016/j.jad.2009.04.028

[ref3] Beekman AT, Geerlings SW, Deeg DJ, Smit JH, Schoevers RS, de Beurs E, Braam AW, Penninx BW, Van Tilburg W (2002). The natural history of late-life depression: a 6-year prospective study in the community. Archives of General Psychiatry 59, 605–611. 12090813 10.1001/archpsyc.59.7.605

[ref4] Bogner HR, Morales KH, Reynolds CF III, Cary MS, Bruce ML (2012). Course of depression and mortality among older primary care patients. American Journal of Geriatric Psychiatry 20, 895–903. 21997603 10.1097/JGP.0b013e3182331104 PMC3262092

[ref5] Boyle VL, Roychoudhury C, Beniak R, Cohn L, Bayer A, Katz I (2004). Recognition and management of depression in skilled-nursing and long-term care settings: evolving targets for quality improvement. American Journal of Geriatric Psychiatry 12, 288–295. 15126230

[ref6] Cohen-Mansfield J, Marx MS, Lipson S, Werner P (1999). Predictors of mortality in nursing home residents. Journal of Clinical Epidemiology 52, 273–280. 10235167 10.1016/s0895-4356(98)00156-5

[ref7] Cox C, Chu H, Schneider MF, Munoz A (2007). Parametric survival analysis and taxonomy of hazard functions for the generalized gamma distribution. Statistics in Medicine 26, 4352–4374. 17342754 10.1002/sim.2836

[ref8] Cuijpers P (2001). Mortality and depressive symptoms in inhabitants of residential homes. International Journal of Geriatric Psychiatry 16, 131–138. 11241717 10.1002/1099-1166(200102)16:2<131::aid-gps283>3.0.co;2-w

[ref9] Cuijpers P, Vogelzangs N, Twisk J, Kleiboer A, Li J, Penninx BW (2013). Differential mortality rates in major and subthreshold depression: meta-analysis of studies that measured both. British Journal of Psychiatry 202, 22–27. 23284149 10.1192/bjp.bp.112.112169

[ref10] Cuijpers P, Vogelzangs N, Twisk J, Kleiboer A, Li J, Penninx BW (2014). Comprehensive meta-analysis of excess mortality in depression in the general community versus patients with specific illnesses. American Journal of Psychiatry 171, 453–462. 24434956 10.1176/appi.ajp.2013.13030325

[ref11] Damián J, Pastor-Barriuso R, Valderrama-Gama E (2010). Descriptive epidemiology of undetected depression in institutionalized older people. Journal of the American Directors Association 11, 312–319. 10.1016/j.jamda.2010.01.012 20511097

[ref12] D'Ath P, Katona P, Mullan E, Evans S, Katona C (1994). Screening, detection and management of depression in elderly primary care attenders. I: the acceptability and performance of the 15 item Geriatric Depression Scale (GDS15) and the development of short versions. Family Practice 11, 260–266. 7843514 10.1093/fampra/11.3.260

[ref13] Drageset J, Eide GE, Ranhoff AH (2013). Anxiety and depression and mortality among cognitively intact nursing home residents with and without a cancer diagnosis: a 5-year follow-up study. Cancer Nursing 36, E68–E74. 10.1097/NCC.0b013e31826fcb11 23051868

[ref14] Fenton J, Raskin A, Gruber-Baldini AL, Menon AS, Zimmerman S, Kaup B, Loreck D, Ruskin PE, Magaziner J (2004). Some predictors of psychiatric consultation in nursing home residents. American Journal of Geriatric Psychiatry 12, 297–304. 15126231

[ref15] Gruber-Baldini AL, Zimmerman SI, Mortimore E, Magaziner J (2000). The validity of the minimum data set in measuring the cognitive impairment of persons admitted to nursing homes. Journal of the American Geriatrics Society 48, 1601–1606. 11129749 10.1111/j.1532-5415.2000.tb03870.x

[ref16] Hartmaier SL, Sloane PD, Guess HA, Koch GG (1994). The MDS Cognition Scale: a valid instrument for identifying and staging nursing home residents with dementia using the Minimum Data Set. Journal of the American Geriatrics Society 42, 1173–1179. 7963204 10.1111/j.1532-5415.1994.tb06984.x

[ref17] Hess KR (1994). Assessing time-by-covariate interactions in proportional hazards regression models using cubic spline functions. Statistics in Medicine 13, 1045–1062. 8073200 10.1002/sim.4780131007

[ref18] Hinton L, Zweifach M, Oishi S, Tang L, Unutzer J (2006). Gender disparities in the treatment of late-life depression: qualitative and quantitative findings from the IMPACT trial. American Journal of Geriatric Psychiatry 14, 884–892. 17001028 10.1097/01.JGP.0000219282.32915.a4

[ref19] Hybels CF, Pieper CF, Blazer DG (2002). Sex differences in the relationship between subthreshold depression and mortality in a community sample of older adults. American Journal of Geriatric Psychiatry 10, 283–291. 11994215

[ref20] Izal M, Montorio I (1993). Adaptation of the Geriatric Depression Scale in Spain: a preliminary study. Clinical Gerontologist 13, 83–91.

[ref21] Jongenelis K, Pot AM, Eisses AM, Gerritsen DL, Derksen M, Beekman AT, Kluiter H, Ribbe MW (2005). Diagnostic accuracy of the original 30-item and shortened versions of the Geriatric Depression Scale in nursing home patients. International Journal of Geriatric Psychiatry 20, 1067–1074. 16250079 10.1002/gps.1398

[ref22] Kane KD, Yochim BP, Lichtenberg PA (2010). Depressive symptoms and cognitive impairment predict all-cause mortality in long-term care residents. Psychology and Aging 25, 446–452. 20545428 10.1037/a0019032 PMC3145972

[ref23] Kiecolt-Glaser JK, Glaser R (2002). Depression and immune function: central pathways to morbidity and mortality. Journal of Psychosomatic Research 53, 873–876. 12377296 10.1016/s0022-3999(02)00309-4

[ref24] Martínez de la Iglesia J, Onís Vilches MC, Dueñas-Herrero R, Albert Colomer C, Aguado Taberné C, Luque Luque R (2002). The Spanish version of the Yesavage abbreviated questionnaire (GDS) to screen depressive dysfunctions in patients older than 65 years. Medifam 12, 620–630.

[ref25] Ministerio de Sanidad Servicios Sociales e Igualdad (2014). Spanish National Death Index. Retrieved 29 June 2014. https://www.msssi.gob.es/en/estadEstudios/estadisticas/estadisticas/estMinisterio/IND_TipoDifusion.htm.

[ref26] Murray J, Banerjee S, Byng R, Tylee A, Bhugra D, Macdonald A (2006). Primary care professionals’ perceptions of depression in older people: a qualitative study. Social Science and Medicine 63, 1363–1373. 16698157 10.1016/j.socscimed.2006.03.037

[ref27] O'Connor BP, Vallerand RJ (1998). Psychological adjustment variables as predictors of mortality among nursing home residents. Psychology and Aging 13, 368–374. 9793113 10.1037//0882-7974.13.3.368

[ref28] Parmelee PA, Katz IR, Lawton MP (1992). Depression and mortality among institutionalized aged. Journal of Gerontology 47, 3–10. 10.1093/geronj/47.1.p3 1730856

[ref29] Pfeiffer E (1975). A short portable mental status questionnaire for the assessment of organic brain deficit in elderly patients. Journal of the American Geriatrics Society 23, 433–441. 1159263 10.1111/j.1532-5415.1975.tb00927.x

[ref30] Rovner BW, German PS, Brant LJ, Clark R, Burton L, Folstein MF (1991). Depression and mortality in nursing homes. Journal of the American Medical Association 265, 993–996. 1992213 10.1001/jama.265.8.993

[ref31] Schulz R, Drayer RA, Rollman BL (2002). Depression as a risk factor for non-suicide mortality in the elderly. Biological Psychiatry 52, 205–225. 12182927 10.1016/s0006-3223(02)01423-3

[ref32] Shah AK, Phongsathorn V, Bielawska C, Katona C (1996). Screening for depression among geriatric inpatients with short versions of the Geriatric Depression Scale. International Journal of Geriatric Psychiatry 11, 915–918.

[ref33] Shah S, Vanclay F, Cooper B (1989). Improving the sensitivity of the Barthel index for stroke rehabilitation. Journal of Clincal Epidemiology 42, 703–709. 10.1016/0895-4356(89)90065-6 2760661

[ref34] Sun W, Schooling CM, Chan WM, Ho KS, Lam TH (2011). The association between depressive symptoms and mortality among Chinese elderly: a Hong Kong cohort study. Journals of Gerontology Series A: Biological Sciences and Medical Sciences 66, 459–466. 10.1093/gerona/glq206 21106705

[ref35] Sutcliffe C, Burns A, Challis D, Mozley CG, Cordingley L, Bagley H, Huxley P (2007). Depressed mood, cognitive impairment, and survival in older people admitted to care homes in England. American Journal of Geriatric Psychiatry 15, 708–715. 17504909 10.1097/JGP.0b013e3180381537

[ref36] Thiebaut AC, Benichou J (2004). Choice of time-scale in Cox's model analysis of epidemiologic cohort data: a simulation study. Statistics in Medicine 23, 3803–3820. 15580597 10.1002/sim.2098

[ref37] White J, Zaninotto P, Walters K, Kivimaki M, Demakakos P, Shankar A, Kumari M, Gallacher J, Batty GD (2015). Severity of depressive symptoms as a predictor of mortality: the English longitudinal study of ageing. Psychological Medicine 45, 2771–2779. 25936473 10.1017/S0033291715000732

[ref38] Wulsin LR, Vaillant GE, Wells VE (1999). A systematic review of the mortality of depression. Psychosomatic Medicine 61, 6–17. 10024062 10.1097/00006842-199901000-00003

