# Association of detected depression and undetected depressive symptoms with long-term mortality in a cohort of institutionalised older people – ERRATUM

**DOI:** 10.1017/S2045796016000342

**Published:** 2016-05-11

**Authors:** J. Damián, R. Pastor-Barriuso, E. Valderrama-Gama, J. de Pedro-Cuesta

In the above named article [1] the details for the Financial Support section are incomplete. The complete details are as follows:

This work was supported by the Spanish Health Research Fund (Fondo de Investigación Sanitaria) (PI96/0201) and the ‘Carlos III’ Institute of Health (Instituto de Salud Carlos III) (PI15CIII/00037).

The authors apologise for this omission.

## References

[ref1] Damián J, Pastor-Barriuso R, Valderrama-Gama E and de Pedro-Cuesta J (2016). Association of detected depression and undetected depressive symptoms with long-term mortality in a cohort of institutionalised older people. Epidemiology and Psychiatric Sciences, available on CJO2016. doi:10.1017/S2045796015001171. PMC6998690 26753838

