# Supplementary material for: Association of detected depression and undetected depressive symptoms with long-term mortality in a cohort of institutionalised older people
Source: Epidemiol Psychiatr Sci. 2016 Jan 12;26(2):189–98. doi: 10.1017/S2045796015001171 (PMC6998690; doi:10.1017/S2045796015001171)
Supplement: Supplementary file 1 [file S2045796015001171sup001.pdf]

APPENDIX. 10-item Geriatric Depression Scale. (D'Ath et al. 1994)

Original version from D'Ath et al. 1994.

DEPRESSION. Then I would ask you some questions about your mood. **In the last <u>last seven days</u>**

1.	Are you basically satisfied with your life?	Yes/NO
2.	Have you dropped many of your activities and interests?	YES/No
3.	Do you feel that your life is empty?	YES/No
4.	Are you afraid that something bad is going to happen to you?	YES/No
5.	Do you feel happy most of the time?	Yes/NO
6.	Do you often feel helpless?	YES/No
7.	Do you feel you have more problems with memory than most?	YES/No
8.	Do you feel full of energy?	Yes/NO
9.	Do you feel that your situation is hopeless?	YES/No
10.	Do you think that most people are better off than you are?	YES/No

Spanish translation used

DEPRESIÓN. A continuación me gustaría hacerle algunas preguntas relacionadas con su estado de ánimo. **En los <u>últimos 7 días</u>**

1.	¿Está usted en términos generales satisfecho con su vida?	1.Si	2. No
2.	¿Ha abandonado muchas de sus actividades e intereses?	1.Si	2. No
3.	¿Siente que su vida está vacía?	1.Si	2. No
4.	¿Teme que algo malo vaya a ocurrirle?	1.Si	2. No
5.	¿Está contento la mayoría del tiempo?	1.Si	2. No
6.	¿Se siente desamparado a menudo?	1.Si	2. No
7.	¿Cree que tiene usted más problemas de memoria que la mayoría?	1.Si	2. No
8.	¿Se siente lleno de vitalidad?	1.Si	2. No
9.	¿Encuentra que su situación es desesperada?	1.Si	2. No
10.	¿Piensa que la mayoría de la gente está mejor que usted?	1.Si	2. No
